# The improvement of agronomic performances in the cold weather conditions for perennial wheatgrass by crossing *Thinopyrum intermedium* with wheat-*Th. intermedium* partial amphiploids

**DOI:** 10.3389/fpls.2023.1207078

**Published:** 2023-10-17

**Authors:** Yizhuo Liu, Weiwei Song, Anning Song, Chunfei Wu, Jiarui Ding, Xiaoning Yu, Jia Song, Miaomiao Liu, Xinyuan Yang, Changtong Jiang, Haibin Zhao, Weifu Song, Dongjun Liu, Xuefeng Yang, Qingjie Song, Xinling Li, Lei Cui, Hongjie Li, Yanming Zhang

**Affiliations:** ^1^ Key Laboratory of Molecular Cytogenetics and Genetic Breeding of Heilongjiang Province, College of Life Science and Technology, Harbin Normal University, Harbin, China; ^2^ Administrative Security Division, Heilongjiang Academy of Agricultural Sciences, Harbin, China; ^3^ Institute of Pratacultural Science, Heilongjiang Academy of Agricultural Sciences, Harbin, China; ^4^ Crop Resources Institute, Heilongjiang Academy of Agriculture Sciences, Harbin, China; ^5^ College of Agriculture, Shanxi Agricultural University, Taiyuan, China; ^6^ National Engineering Laboratory for Crop Molecular Breeding/Institute of Crop Sciences, Chinese Academy of Agricultural Sciences, Beijing, China

**Keywords:** *Thinopyrum intermedium*, wheatgrass, chromosome, sequential multicolor GISH, subgenome

## Abstract

*Thinopyrum intermedium* (2n=6x=42, StStJ^r^J^r^J^vs^J^vs^) is resistant or tolerant to biotic and abiotic stresses, making it suitable for developing perennial crops and forage. Through five cycles of selection, we developed 24 perennial wheatgrass lines, designated 19HSC-Q and 20HSC-Z, by crossing wheat-*Th. intermedium* partial amphiploids with *Th. intermedium*. The cold resistance, morphological performance, chromosome composition, and yield components of these perennial lines were investigated from 2019 to 2022. Six lines of 19HSC-Q had higher 1,000-kernel weight, grains per spike, and tiller number than *Th. intermedium*, as well as surviving -30°C in winter. Lines 19HSC-Q14, 19HSC-Q18, and 19HSC-Q20 had the best performances for grain number per spike and 1,000-kernel weight. The 20HSC-Z lines, 20HSC-Z1, 20HSC-Z2, and 20HSC-Z3, were able to survive in the cold winter in Harbin and had been grown for two years. Sequential multicolor GISH analysis revealed that the J^vs^ subgenome of *Th. intermedium* were divided into two karyotypes, three pairs of type-I J^vs^ chromosomes and four pairs of type-II J^vs^ chromosomes. Both *Th. intermedium* and the 24 advanced perennial wheatgrass lines had similar chromosome compositions, but the translocations among subgenome chromosomes were detected in some lines with prominent agronomic traits, such as 19HSC-Q11, 19HSC-Q14, 19HSC-Q18, 19HSC-Q20, and the three 20HSC-Z lines. The chromosome aberrations were distinguished into two types: the large fragment translocation with St-J^r^, J^vs^-St, J^r^-IIJ^vs^, and J^vs^-J^r^ and the small fragment introgression of J^r^-St, St-IJ^vs,^ and J^vs^-J^r^. These chromosomal variations can be used to further analyze the relationship between the subgenomes and phenotypes of *Th. intermedium*. The results of this study provide valuable materials for the next selection cycle of cold-resistant perennial wheatgrass.

## Introduction

The continuous growth of the global population increases the demand for food and livestock. Thus, plenty of lands that are most suitable for annual crops have already been used. The competition for arable lands between non-food products (e.g., biofuels) and food crops is becoming more and more fierce (Glover et al., 2010; Godfray et al., 2010; Springmann et al., 2018). Although current farming methods have enabled the achievement of unprecedented yields, they have also exacerbated ecosystem problems, such as soil erosion, greenhouse gas emissions, and water pollution (Monfreda et al., 2008). The strategy of the “ecological intensification of agriculture” was proposed to enhance food production with expanded ecosystem services and reduced inputs (DeHaan et al., 2018). Many studies have demonstrated that perennial crops are useful to intensify agricultural ecology because they have advantages over annual crops in soil carbon balance, nutrient retention, soil water uptake efficiency, functional utilization of soil microbiomes, and weed suppression (Cox et al., 2006; Batello et al., 2013; DeHaan, 2015; Kantar et al., 2016; Crews and Cattani, 2018; Ryan et al., 2018). Therefore, it is a common objective for breeding for perennial crops with acceptable grain yields without environmental penalties.

*Thinopyrum intermedium* (Host) Barkworth and Dewey (2n=6x=42, StStJ^r^J^r^J^S^J^S^), also known as intermediate wheatgrass (IWG), is a perennial grass in the tribe Triticeae. It has lush plant growth, high stature and leaf protein content, cold-hardiness, and tolerance to drought and salt stresses (Wagoner, 1995; Harmoney, 2015). This perennial grass species has been used extensively as a cool-season forage in the USA, Canada, and across its native range in Eurasia (Wagoner, 1995). Because of its developed perennial rhizome system, IWG improves the qualities of soil and surface and ground water, provides a habitat for wild animals (such as Waterfowl nesting sites), isolates carbon sources in the atmosphere, and impacts ecosystem improvement (DeHaan and Ismail, 2017; DeHaan et al., 2018). Moreover, IWG was initially selected as a target species for domestication of a perennial grain because it has synchronous seed maturity, edible grain, moderate shattering, and reasonable thresh ability; it also has higher contents of grain protein and fiber than common wheat (*Triticum aestivum* L.) (Wagoner, 1990; Wagoner, 1995). Grains of IWG have similar nutritional compositions to common wheat, in spite of differences in the protein properties of the flour. The flour of IWG can be mixed with common wheat flour in an appropriate proportion to make baking products (Becker et al., 1991; Zhang et al., 2014; Zhang et al., 2015; Marti et al., 2016).

The Rodale Research Center at Kutztown, PA, USA, has initiated a project to domesticate *Th. intermedium* aiming at improving its yield components and perenniality since 1983 (Wagoner, 1990). From 1991 to 1995, the Rodale Research Center and Big Flats Plant Materials Center (USDA) completed two cycles of selection for IWG, and the average grain yield per plant was 25% higher than that of the whole population (Wagoner and Schaeffer, 1990; Wagoner, 1995). Subsequently, scientists at the Land Institute (TLI), Saline, KS, USA, have worked on some of the selected plants for improving yield per spike, seed size, free threshing, reduced plant height, and early maturity since 2003 (DeHaan et al., 2018; Bajgain et al., 2020). The first IWG cultivar, Kernza^®^, was commercialized after nine cycles of selection (DeHaan et al., 2018; Bajgain et al., 2020). In 2011, the University of Manitoba, Winnipeg, Canada, and the University of Minnesota, St. Paul, USA, also initiated projects to directly domesticate wheatgrass germplasm from TLI (Cattani, 2016; Zhang et al., 2016). The first commercial food-grade IWG cultivar MN-Clearwater (Reg. no. CV-287, PI 692651) was released as a synthetic population by the University of Minnesota in 2019 (Bajgain et al., 2020). In the variety trials across Minnesota, MN-Clearwater produced 696 kg ha^−1^ of grains with minimal lodging and negligible levels of diseases (Bajgain et al., 2020). An IWG breeding program was also initiated in Utah, USA, and Uppsala, Sweden (Bajgain et al., 2020).

As an allohexaploid species, *Th. intermedium* carries three subgenomes with chromosomes sorted in seven homoeologous groups. The genome composition of *Th. intermedium* has been extensively studied with molecular cytogenetic methods leading to different hypotheses (Chen et al., 1998; Chen, 2005; Mahelka et al., 2011; Mahelka et al., 2013; Li et al., 2016). Using EST-SSR primers developed from the putative progenitor diploid species *Pseudoroegneria strigosa* (Pursh) (2n=2x=14, StSt), *Th. bessarabicum* (Savul. & Rayss) (2n=2x=14, JJ=J^b^J^b^), and *Th. elongatum* (Host) (2n=2x=14, EE=J^e^J^e^), *Th. intermedium* is proposed to have been formed by an ancient hybridization event between the diploid species *Ps. strigosa* possessing the St genome and a segmental tetraploid species carrying the J^r^ and J^vs^ genomes (Wang et al., 2015). The J^r^ and J^vs^ genomes represent ancestral genomes from *Th. bessarabicum* (J^b^) and *Th. elongatum* (J^e^), respectively. Genome J^vs^ is distinct from genome J^b^ as it retains repetitive sequences from the V genome of *Dasypyrum villosum* (L.) P. Candargy, while J^r^ carries a long terminal repeat originating from the R genome of rye (*Secale cereale* L., 2n=2x=14, RR) (Kishii et al., 2005; Mahelka et al., 2011; Wang et al., 2015). Cseh et al. (2019) suggested that the *Th. intermedium* chromosomes originated from three genomes of *Ps. strigosa*, *Th. bessarabicum*, and *D. villosum*, and the genome composition of *Th. intermedium* was proposed to be StStJ^r^J^r^J^vs^J^vs^.

Currently, the direct domestication strategy based on recurrent phenotypic selection is widely used in most breeding programs for the improvement of *Th. intermedium*. This method relies on plant variations within a population. DeHaan et al. (2014) suggested that it is possible to develop perennial grain crops from wheat with grass-like traits or perennial grass with wheat-like traits. It is less difficult to introduce domesticated genes from common wheat into perennial plants to improve their wild traits, such as seed and spike shattering, infinite flowering, and small kernels (Abbo et al., 2014). This offers a new strategy to accelerate the domestication of *Th. intermedium*.

The climate of Heilongjiang province in northeastern China is mainly characterized by cold and dry winters, and especially cold spells in later spring. Degradation, desertification, and salinization are expanding in the areas of grassland in this province. The improvement of grasslands urgently requires forage varieties with strong cold tolerance and wide adaptability. To obtain perennial forages, cold tolerance is a priority in this frigid zone. Despite *Th. intermedium* incorporating cold tolerance and perennial growth, the properties of small grains, low seed setting rate, and insufficient yield has limited its commercialization in agriculture. Compared to IWG, common wheat has better yield traits that can be used for improving IWG yield through distance hybridization. However, it is difficult to obtain stabilized hybrid progenies between common wheat and IWG due to substantial differences in their genomic structures and compositions. Octaploid trititrigia (2n=8x=56), carrying both wheat chromosomes and partial *Thinopyrum* chromosomes, is likely to make the chromosomal balance for the newly formed wheatgrasses after hybridization with IWG.

We developed 34 perennial wheatgrass lines with vigorous growth and cold-hardiness by recurrent phenotypic selection from the cross between octoploid trititrigia and *Th. intermedium* (Yan et al., 2020). Twenty-four perennial progeny lines with desirable agronomic performances were selected after five cycles of selection from 2007 to 2022. One of the objectives of this study was to analyze the morphological performances of these perennial lines with cold-hardy tests and improve the potential in grain production; another was to characterize their chromosome compositions using sequential multicolor genomic *in situ* hybridization (smGISH) for determining their subgenome chromosomal variations. The long-term aim of this project is to breed new perennial forage wheatgrasses adapted to the local winter hardy environments in northeast China.

## Materials and methods

### Plant materials

The *Th. intermedium* accession used was provided by the Heilongjiang Academy of Agricultural Sciences, Harbin, Heilongjiang, China. In 2006, it was crossed as the paternal parent with annual wheat-*Th. intermedium* partial amphiploids Maicao 8 (919-C/Yuan16-3) and Maicao 9 (2A2/Xiaoyan 68) as the maternal parents. Yuan16-3 and Xiayan 68 are wheat-*Th. intermedium* and wheat-*Th. ponticum* partial amphiploids developed by the Shanxi Academy of Agricultural Sciences, Taiyuan, Shanxi, China, and the Northwest Institute of Botany, Chinese Academy of Science, Yangling, Shanxi, China, respectively. Twenty perennial wheatgrass lines were obtained after two rounds of three-year artificial and natural selection based on the recurrent phenotypic evaluation (HNU-C1 and HNU-C2) in the field of Harbin Normal University, Harbin, Heilongjiang (126°57’E, 45°87’N) from 2007 to 2013 (Yan et al., 2020). From 2013 to 2022, another three cycles of selection for perennial wheatgrass lines, HNU-C3, HNU-C4, and HNU-C5, were carried out under the natural cold winter conditions. Accessions of *Ps. strigosa*, *Th. bessarabicum*, and *D. villosum* were used as sources of DNA to prepare probes for the detection of St-, J^b^-, and V-genome chromosomes or chromosomal segments in the IWG lines.

### Evaluation of perennial cold-hardiness and agronomic performances

**Figure 1:**
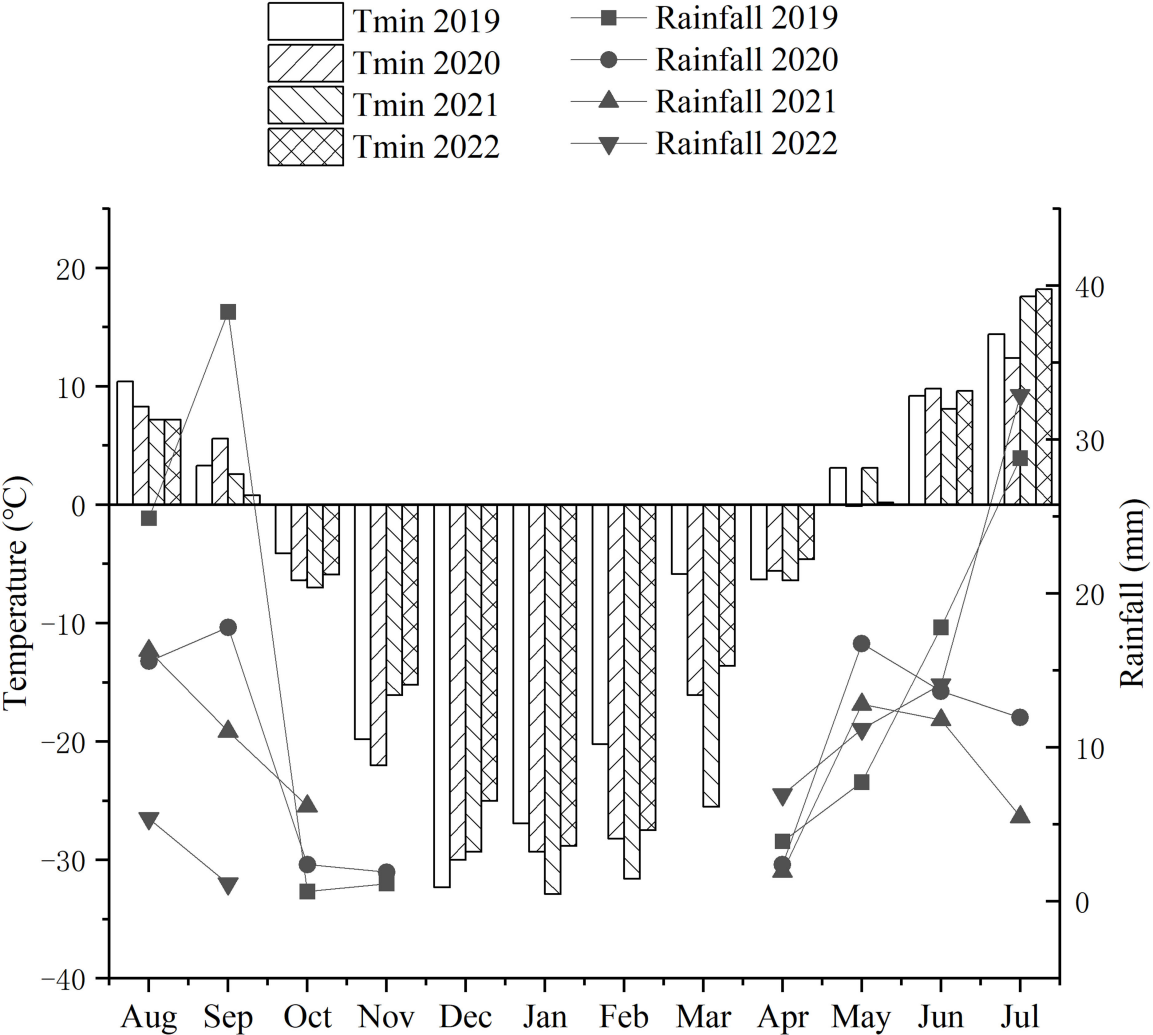
Monthly precipitation (mm) and minimum (Tmin) temperatures during the growth cycles of each growing season from 2019 to 2022. From October to April of each year between 2019 and 2022, the temperatures of the winter season in Harbin remained below freezing point. During the domestication breeding of the cold tolerance of perennial wheatgrass, the average temperature ranged from -16.5°C to -21.26°C for the four consecutive years, and the lowest temperature experienced during the cold resistance test was -32.9°C in January 2021. The average precipitation in the four years was in the range of 5-18 mm.

From 2007, the newly developed IWG lines were investigated in an experimental farm of Harbin Normal University, Harbin, China. The agricultural climate of Harbin is warm semiarid, with spring drought and semi-humid summers. From October to April of each year during 2019-2022, the temperature of the winter season in Harbin was below freezing point. During the domestication breeding for cold tolerance of perennial wheatgrasses, the average temperature ranged from -16.5°C and -21.26°C for the four consecutive years, with the lowest temperature of -32.9°C in January 2021 (**Figure 1**).

In autumn of 2019 and 2020, perennial wheatgrass lines were sown in the experimental field plots for HNU-5C. The field management, seeding, and agronomic characterization were carried out as described previously (Yan et al., 2020). Defoliation was performed in August for harvesting both grains and forages each year. Post-harvest regrowth of aboveground parts and underground rhizomes were observed from harvest to the onset of winter in October and November. From November to early April in the following year, the plants in fields were exposed to the natural cold environments to examine their cold-hardiness based on their survivability during winter and regrowth in spring. Plants that were able to regrow for at least three consecutive years in fields were regarded as cold-hardy perennials. Maicao 8, Maicao 9, and the *Th. intermedium* accession were grown in 1m rows and 0.3 m spacing between rows as the annual and perennial controls, respectively.

During the growing seasons, plant height (cm), tiller number, spike length (cm), seed set percentage, grain number per spike, and 1,000-kernal weight (g) were investigated for the perennial wheatgrass lines. Plant height was determined from the ground level to the top of a spike. Spike length was measured from the base of a rachis to the top of a spike. The numbers of kernels and spikelets per spike were enumerated and spikes were threshed in a bench micro-thresher to determine the 1,000-kernel weight. Common practices of irrigation (one before the overwinter stage immediately after defoliation in November, and the other after grain and forage harvesting in July), fertilizers (nitrogen, phosphorus, and potassium), and pesticides were applied following the local farming system (Yan et al., 2020).

### Chromosome preparation

Seeds were germinated at 23.5°C for 24 h on moist filter paper in Petri dishes prior to incubation at 4°C for 48 h and 23.5°C for 27.5 h. Root tips were treated with ice water at 0-4°C for 24 h, fixed in Carnoy’s fixative (anhydrous alcohol: acetic acid = 3:1, v/v) for 24 h, and squashed in 45% acetic acid. Chromosome preparations were observed under a phase contrast microscope (BH-2, Olympus, Tokyo, Japan).

### Sequential multicolor GISH

Genomic DNA was isolated from young leaves of *Ps. strigosa*, *Th. bessarabicum*, and *D. villosum* to prepare the St-, J^b^-, and V-genome probes using the cetyltrimethylammonium bromide (CTAB) method (Doyle and Doyle, 1987). Five seeds randomly selected from each line were subjected to smGISH analysis. In the first round of GISH, the genomic DNA of *Ps. strigosa* and *Th. bessarabicum* were separately labeled with fluorescein-12-dUTP (green) (BioNick Labeling System; Invitrogen, Waltham, MA, USA) and tetranmethyl-rhodamine-5-dUTP (red) (DIG-Nick Translation Mix, Roche Diagnostics, Mannheim, Germany). In the second round of GISH, the genomic DNA of *D. villosum* and *Ps. strigosa* were separately labeled with fluorescein-12-dUTP (green) and tetranmethyl-rhodamine-5-dUTP (red). The protocol of smGISH was described previously by Yan et al. (2020) and Cseh et al. (2019). All slides were visualized using a Leica DM5500B epifluorescence microscope (Leica Microsystems, Wetzlar, Germany) equipped with different filters for detecting DAPI [2-(4-Amidinophenyl)-6-indolecarbamidine dihydrochloride] (blue), Alexa Fluor 488 (green), and Alexa Fluor 594 (red). A Leica DM6000B fluorescence microscope (Leica, Mannheim, Germany) was used for observing the hybridization signals, and photographs were captured with a Leica digital camera (Model DFC480) and processed by Photoshop 8.0 (Adobe, San Jose, California, USA).

### Statistical analysis

Values of the agronomic traits investigated were subjected to analysis of variance (ANOVA) using genotype, year, and genotype by year interaction as the main factors. Fisher’s least significant difference (LSD) was used to perform multiple comparisons for differentiating the significant differences among genotypes and plant ages (i.e., the first, second, and third-year plants) based on the means of each parameter examined. Statistical analysis was performed using IBM SPSS Statistics for Windows Version 19 (SPSS Inc., Chicago, IL, USA). Significant differences in the growth years and various traits of different materials were identified by the *t*-test of multiple samples at *P* < 0.05.

### Protein content of measurement

Four-hundred seeds of each line with the same seed shape were placed into the sample cup. A DA7200 multifunctional near infrared spectrometer (Perten, Switzerland) was used for testing seed protein content. Each line was tested three times.

## Results

### Selection for the perennial wheatgrass lines

**Figure 2:**
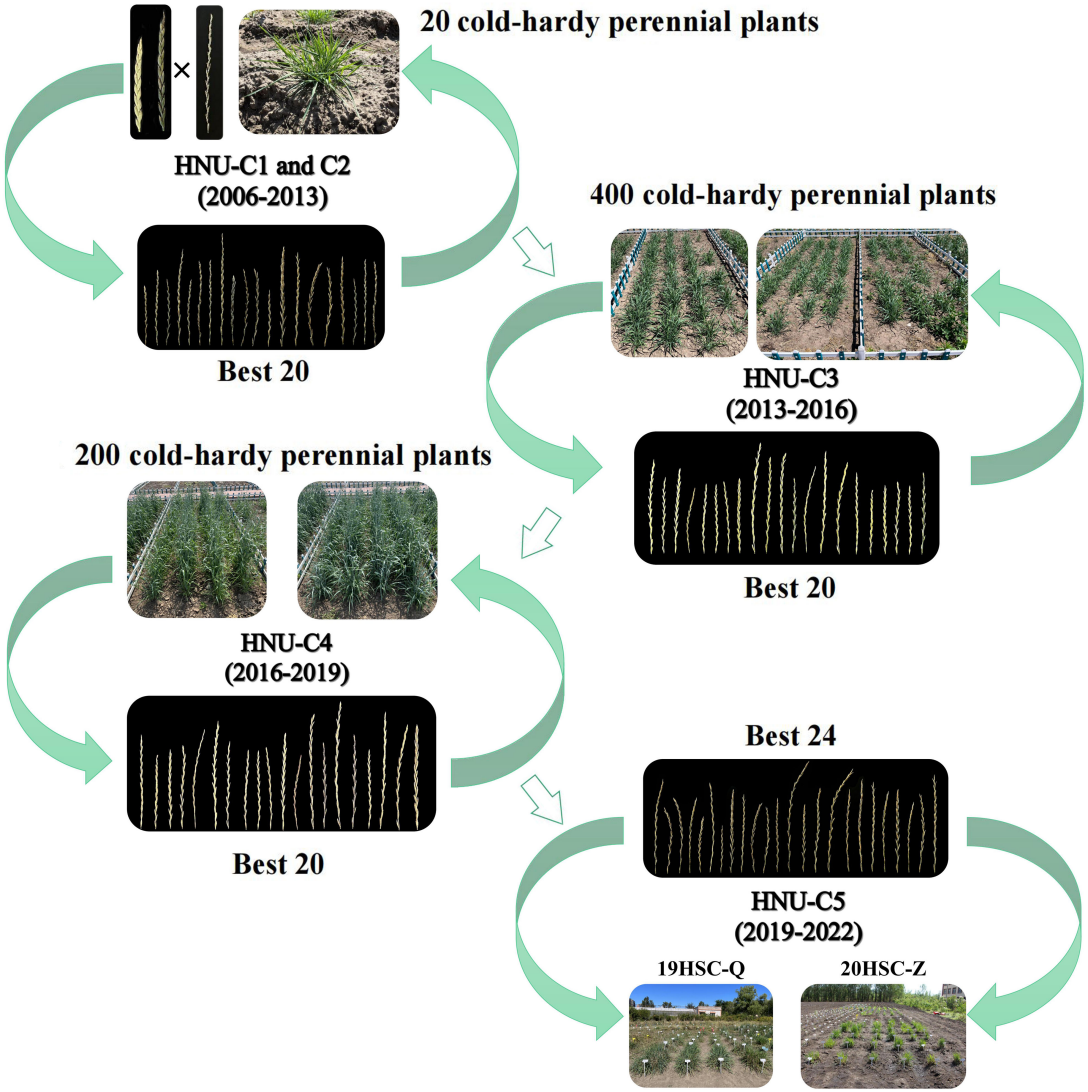
Breeding process of new germplasm of *Thinopyrum intermedium*. The new IWG germplasms were developed through the cross breeding of octoploid trititrigia × *Th. intermedium* from 2006. From 2007 to 2022, five cycles of selection, each with 3-year as a selection cycle, were carried out in Harbin Normal University under the cold winter climate. In 2022, two populations named 19HSC-Q and 20HSC-Z were selected for molecular cytogenetic analysis.

From 2013 to 2022, three cycles of selection were performed under natural field conditions (**Figure 1**). Plants without cold tolerance and/or weak growth ability were eliminated by the cold winter in Harbin. Four-hundred cold-hardy perennial plants were obtained during the third cycle of selection (HNU-C3) from 2013 to 2016. Twenty plants were selected because of their high seed setting, cold tolerance, and strong post-harvest regrowth ability. In the fourth cycle of selection (HNU-C4) from 2016 to 2019, 200 perennial plants were selected. Finally, 24 perennial cold-hardy wheatgrass lines, designated 19HSC-Q and 20HSC-Z, were developed in 2019 and 2020. From 2019 to 2022, as HNU-C5, twenty-one 19HSC-Q and three 20HSC-Z lines were subjected to cold-hardiness tests and molecular cytogenetic analysis (**Figure 2**).

### Cold-hardiness and maturity of the perennial wheatgrass lines

**Figure 3:**
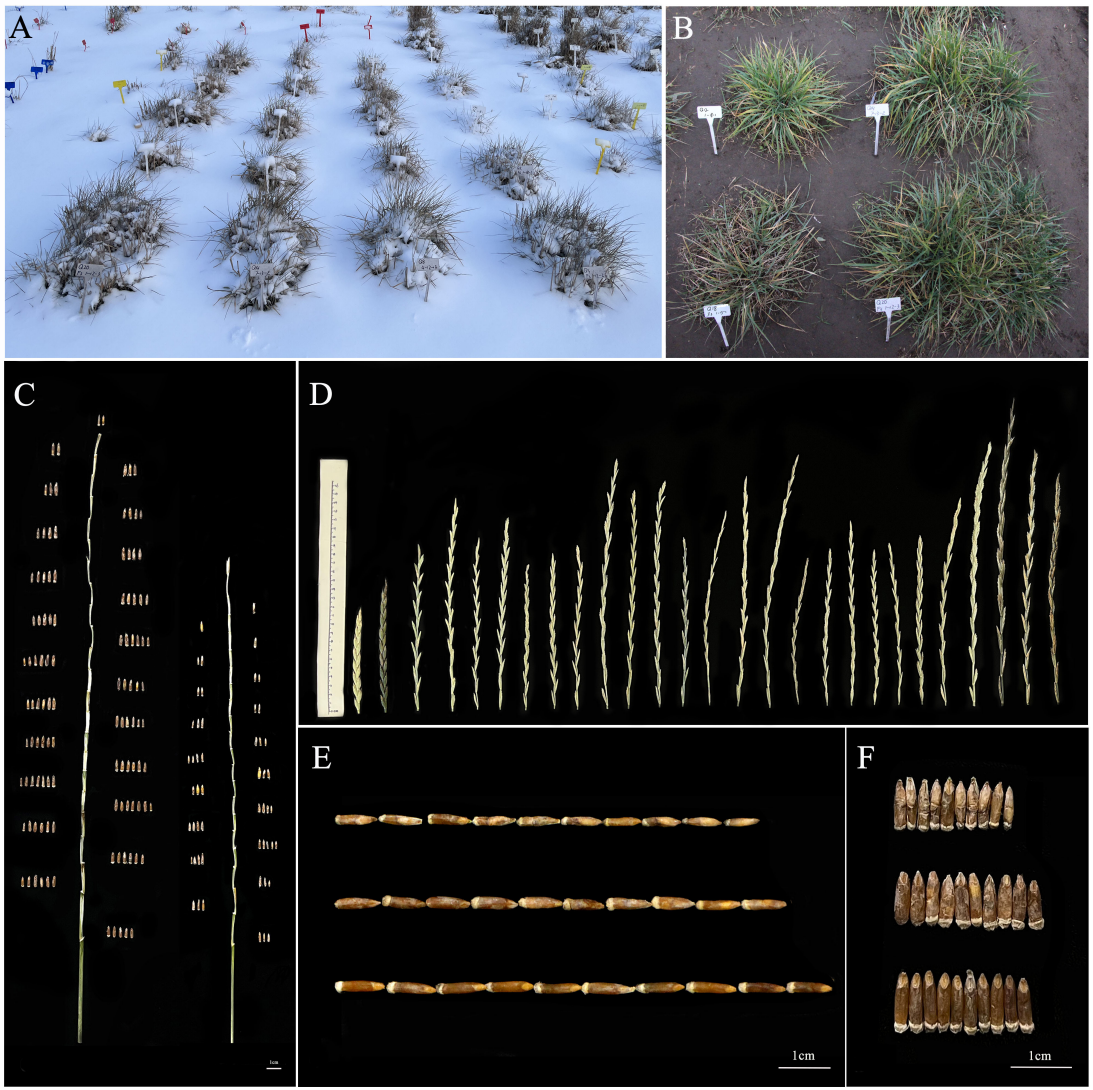
The observation of plant growth and morphological characteristics of spike and grain. **(A)** Lines of 19HSC-Q were covered with snow (photographed in November 2022). **(B)** The cluster area of 19HSC-Q12 (top left), 19HSC-Q14 (top right), 19HSC-Q18 (bottom left), and 19HSC-Q20 (bottom right) (photographed in October 2022). **(C)** Grain number per spike of 19HSC-Q14 (left) and IWG (right). **(D)** Spikes of the wheatgrass plants from 19HSC-Q to 20HSC-Z generations and their parents. The first panel from left to right shows Maicao 8, Maicao 9, and *Th. intermedium*, followed by lines 19HSC-Q from 19HSC-Q2 to 19HSC-Q23. The last three are 20HSC-Z1, 20HSC-Z2, and 20HSC-Z3 (photographed in July 2022). **(E, F)** Seed characteristics of perennial wheatgrasses. Grain lengths **(E)** and grain widths **(F)** of IWG, 19HSC-Q14, and 20HSC-Z1 from the top to the bottom, respectively. Scale bars = 1 cm.

From 2019 to 2022, twenty-one 19HSC-Q perennial lines were evaluated for their cold hardiness, perennial habit, and maturity. All of them survived for more than 3 years, showing perennial growth habit, which resembled their perennial parent *Th. intermedium*. The coleoptile of these lines was purple. The seedlings stopped growing and were dormant during snow-cover in the natural cold winter, but the leaves and stems remained green during the whole winter. In 2020, all the 19HSC-Q lines resumed normal growth in April, flowered for a week from 22 June, and matured by the end of July. Post-harvest regeneration was observed after cutting the aboveground parts in early August 2020. From September to October, new stems and leaves emerged from crowns or roots, and the regenerated plants grew vigorously. The growth of lines slowed down in November and entered a dormant period after snow-cover in December (**Figure 3A**). From November 2020 to the end of March 2021, some stems and leaves around the crowns remained green in all the regenerated lines, and the new rhizomes appeared in the soil. In April 2021, the leaves and stems were withered. After cutting, new leaves regrew from the original crowns and the lines returned to the vegetative state for a new growth cycle in May 2021. The plants resumed growth again, and the underground rhizomes developed into new plants. The rates of returning green was 100% for all the 19HSC-Q lines. The subsequent performance of the plants was consistent with the first growth cycle. All lines were harvested at the end of July 2021. The 19HSC-Q lines still maintained the post-harvest regeneration. In the third growth cycle from 2021 to 2022, the plants kept a stable rate of returning green and regeneration after cutting (**Figure 3B**), and the dates to flowering and maturity were consistent with those of the previous two growth years. Lines 19HSC-Q5 and 19HSC-Q17 also were cold-tolerant, but the vitality of plants was weak. The ability of regeneration and returning green were decreased also, and the crowns were gradually shrunk. Currently, all the 19HSC-Q lines are experiencing their fourth growth cycle.

From 2020 to 2022, three 20HSC-Z lines in HNU-5C were tested for cold tolerance and maturity in the natural cold winter. These cold-hardy perennial lines had post-harvest regrowth and their growth habits were consistent with other 19HSC-Q lines. At present, they have entered their third growth cycle.

All the 19HSC-Q and 20HSC-Z perennial wheatgrass lines with cold tolerance and post-harvest regeneration (HPR) were seeded in Harbin during 2019 and 2020. Compared to the previous four selection cycles, dates to flowering and maturity of the 19HSC-Q plants were about 40 days earlier. It is more adapted to the cold winter of Harbin.

### Performances of morphological and yield traits

The agronomic traits of twenty-one 19HSC-Q perennial lines were investigated for three consecutive growing seasons from 2019 to 2022. Except 19HSC-Q5 and 19HSC-Q17, all lines maintained the wheatgrass characteristics in plant height, heading date, and tillering ability. The agronomic performances of these lines varied and the land cover area per plant was inconsistent after three growth cycles.

**Figure 4:**
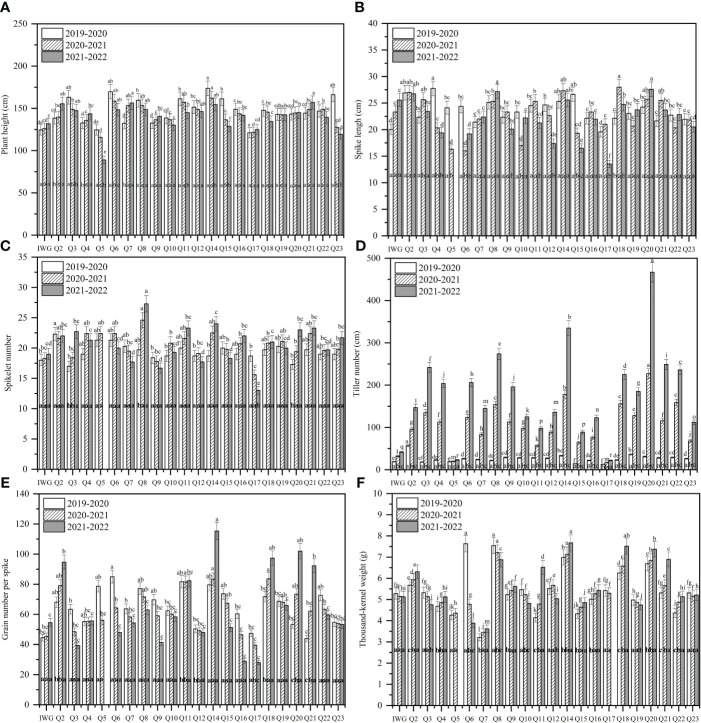
Assessment of agronomist traits of plant height **(A)**, spike length **(B)**, spikelet number **(C)**, tiller number **(D)**, grain number per spike **(E)**, and 1,000-kernel weight **(F)** for the perennial wheatgrass lines developed and the control IWG over the three growing seasons 2019-2020, 2020-2021, and 2021-2022 in Harbin. For each trait per growing season, different letters inside bars indicate a significant difference among lines using Fisher’s least significant difference (LSD) (*P* < 0.05). For the tested variables of each line, different letters above bars indicate a significant difference among growing seasons (*P* < 0.05).

The average plant height of 19HSC-Q lines over 3 years ranged from 139.9 cm to 147.6 cm (**Figure 4A**). The average spike length ranged from 22.35 cm to 26.92 cm (**Figures 3D**, **4B**). The average number of spikelets ranged from 19.37 to 21.11 with four to five pairs of florets per spikelet, and the highest number of 24 spikelets was observed in 19HSC-Q14 in 2022 (**Figure 4C**; **Table 1**). The average spike length and spikelet number per spike of 19HSC-Q5, 19HSC-Q7, 19HSC-Q9, 19HSC-Q12, 19HSC-Q15, and 19HSC-Q17 were lower than those of the control *Th. intermedium*. Although 19HSC-Q5 and 19HSC-Q17 have grown for three years, 19HSC-Q5 was sterile and 19HSC-Q17 produced only a few seeds in 2022.

**Table 1:** Agronomic traits, chromosome karyotype, and protein content for nine cold-hardiness perennial wheatgrass lines and IWG in 2022.

Lines	Years of growth	Chromosome karyotype	Plant height (cm)	Spike length (cm)	Spikelet number	Tiller number	Grain number per spike	1,000-kernel weight (g)	Seed setting rate (%)	The land cover area per plant (m^2^)	Seed length (mm)	Seed width (mm)	Protein content (%)
IWG	3	42 = 14St+14J^r^+6IJ^vs^+8IIJ^vs^	131.8	19.41	17	102	54.6	5.128	42.68	0.093	3.415	0.918	15.47
19HSC-Q2	3	42 = 14St+14J^r^+6IJ^vs^+8IIJ^vs^	155.4	26.75	22	147	94.67	6.314	52.9	0.267	4.572	1.355	17.21
19HSC-Q11	3	42 = 14St+14J^r^+5IJ^vs^+8IIJ^vs^+1St-IJ^vs^	144.9	21.27	23.3	98	82.33	6.521	43.0	0.204	4.128	1.218	17.11
19HSC-Q14	3	42 = 13St+14J^r^+6IJ^vs^+8IIJ^vs^+1J^r^-St	154.6	25.58	24	335	115	7.674	50.3	0.335	5.535	1.472	17.36
19HSC-Q18	3	42 = 13St+14J^r^+6IJ^vs^+8IIJ^vs^+1V-St	134.4	24.76	21	225	97.33	7.516	55.9	0.272	4.584	1.246	19.14
19HSC-Q20	3	42 = 12St+13J^r^+6IJ^vs^+8IIJ^vs^+1J^r^-IIJ^vs^+2J^r^-St	145.1	23.73	23	467	102	7.371	54.5	0.615	4.657	1.347	19.89
19HSC-Q21	3	42 = 14St+14J^r^+6IJ^vs^+8IIJ^vs^	156.9	27.61	23.3	249	92.33	6.895	43.5	0.247	4.697	1.245	17.73
20HSC-Z1	2	42 = 13St+13J^r^+6IJ^vs^+8IIJ^vs^1J^r^-IIJ^vs^+1J^r^-St	155	27.97	21	131	116	7.146	52.46	0.058	6.314	1.218	20.01
20HSC-Z2	2	42 = 13St+14J^r^+6IJ^vs^+8IIJ^vs^+1J^r^-St	133.3	27.17	24	149	88.67	6.523	52.94	0.119	4.346	1.365	17.85
20HSC-Z3	2	42 = 13St+14J^r^+6IJ^vs^+8IIJ^vs^+1J^r^-St	132.7	26.41	19	173	81	5.916	55.69	0.151	3.728	1.146	18.52

The tiller number of 19HSC-Q lines increased over the years. For example, line 19HSC-Q2 produced 57 tillers in the first growth cycle, while all the other lines had 14 to 38 tillers. In the third growth year, the tiller number of 19HSC-Q2 was 147. Lines 19HSC-Q14 (335) and 19HSC-Q20 produced 335 and 467 tillers, respectively. The tiller numbers of 19HSC-Q14 and 19HSC-Q20 in the first life year were only 33 and 31. In the third growth cycle, there were nine lines with more than 200 tillers (**Figure 4D**). In 2022, the lines with the largest number of tillers was 19HSC-Q20, which also had the largest land cover area per plant (0.615 m^2^), followed by 19HSC-Q14 (0.335 m^2^) and 19HSC-Q8 (0.272 m^2^). The other lines ranged from 0.080 m^2^ to 0.615 m^2^ for the land cover area per plant.

The agronomic characteristics of three 20HSC-Z lines were measured from 2020 to 2022 (**Table 1**). The average plant height ranged from 55 cm to 138 cm in the two growth cycles. The spike length and the number of spikelets per spike ranged from 18.37 cm to 27.97 cm and 19 to 24, respectively. The number of florets per spike were four to six pairs. In 2022, the plant height and spike length of 20HSC-Z1 were 155 cm and 27.97 cm, respectively (**Figure 3D**). The highest number of spikelets was 24 for 20HSC-Z2. In 2022, the largest tiller number of the three 20HSC-Z lines was 173 for 20HSC-Z3. The largest land cover area per plant was also observed in 20HSC-Z3 (0.151 m^2^), followed by 20HSC-Z2 (0.119 m^2^) and 20HSC-Z1 (0.058 m^2^).

During 2019-2022, the number of grains per spike and 1,000-kernal weight varied significantly among the 21 19HSC-Q lines, and their grain protein content was all greater than 15%. In the first life year, the number of grains per spike of 15 lines exceeded 60, but 14 of them decreased each year. Seven lines increased in the number of grains per spike during the three growth cycles. In the third growth cycle, lines 19HSC-Q2, 19HSC-Q11, 19HSC-Q14, 19HSC-Q18, 19HSC-Q20, and 19HSC-Q21 had more than 80 grains per spike in comparison with 54.6 of *Th. intermedium* (**Figure 4E**; **Table 1**). The grain numbers per spike of the top three lines 19HSC-Q14, 19HSC-Q20, and 19HSC-Q18 in 2022 were 115, 102, and 97, respectively (**Figures 3C**, **4E**; **Table 1**). The average seed set for these lines was around 50%, and the 1,000-kernal weight in 2022 was more than 6 g, which was higher than that of IWG (5.128 g). Lines 19HSC-Q14, 19HSC-Q18, and 19HSC-Q20, with the highest grain numbers per spike, also had a 1,000-kernal weight of 7.67, 7.52, and 7.37 g, respectively (**Figure 4F**; **Table 1**). In 2022, the average grain length and width of the six lines with greater number of grains per spike were 4.13-5.54 mm and 1.22-1.47 mm, which were higher than those of the IWG (3.42 mm and 0.92 mm, respectively). The largest average length (5.54 mm) and width (1.47 mm) of grains were observed in line 19HSC-Q14 (**Figures 3E, F**; **Table 1**). The protein content of 10 lines was more than 17%, and 19HSC-Q14, 19HSC-Q18, and 19HSC-Q20, with the best grains per spike and 1,000-kernal weight, had protein contents of 17.36%, 19.14%, and 19.89%, respectively (**Table 1**).

In 2022, the numbers of grains per spike of the three 20HSC-Z lines were all greater than 80, and the seed setting rates were over 50%. The highest number of grains per spike was 116 for 20HSC-Z1. 20HSC-Z3 had the highest setting rate of 55.69% (**Table 1**). The 1,000-kernal weight of 20HSC-Z1, 20HSC-Z2, and 20HSC-Z3 were 7.146, 6.523 g, and 5.916 g, respectively. The grain length of these lines ranged from 3.728 mm to 6.314 mm, and the average width ranged from 1.146 mm to 1.365 mm (**Figure 3E**; **Table 1**). The protein contents of these three lines were more than 17% protein, with the highest of 20.01% for and 20HSC-Z1.

**Figure 5:**
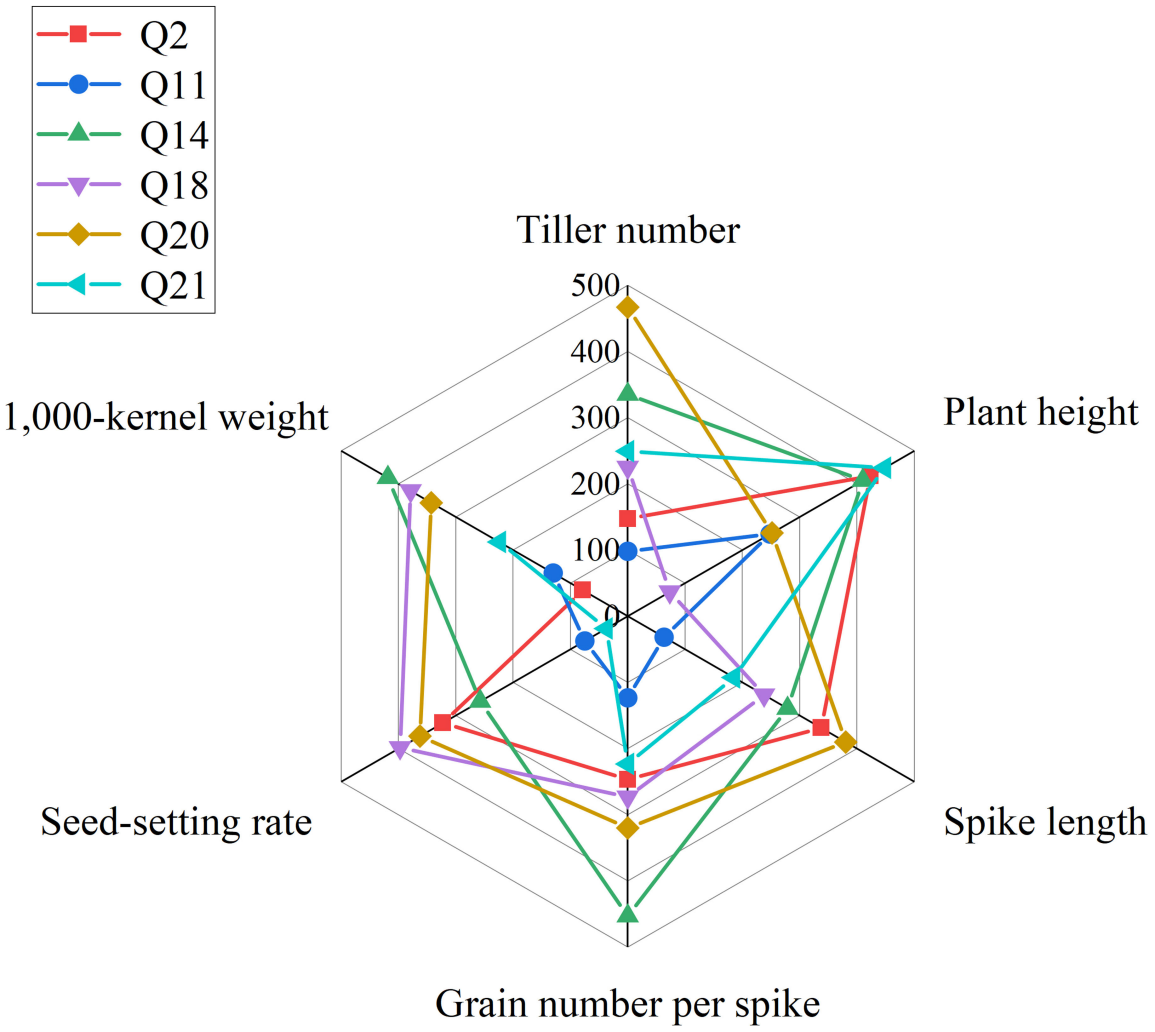
Radar map of agronomic traits of plant height, spike length, tiller number, grain number per spike, 1,000-kernal weight, and seed-setting rate. The red, blue, green, purple, brown, and yellow lines represent 19HSC-Q2, 19HSC-Q11, 19HSC-Q14, 19HSC-Q18, 19HSC-Q20, and 19HSC-Q21, respectively.

In summary, we obtained six lines, 19HSC-Q2, 19HSC-Q11, 19HSC-Q14, 19HSC-Q18, 19HSC-Q20, and 19HSC-Q21, with the most outstanding agronomic traits and yield components in HNU-5C. Lines 19HSC-Q14, 19HSC-Q18, and 19HSC-Q20 had the best agronomic traits over the other perennial lines (**Figure 5**).

### Sequential multicolor GISH analysis

#### SmGISH of *Th. intermedium*

**Figure 6:**
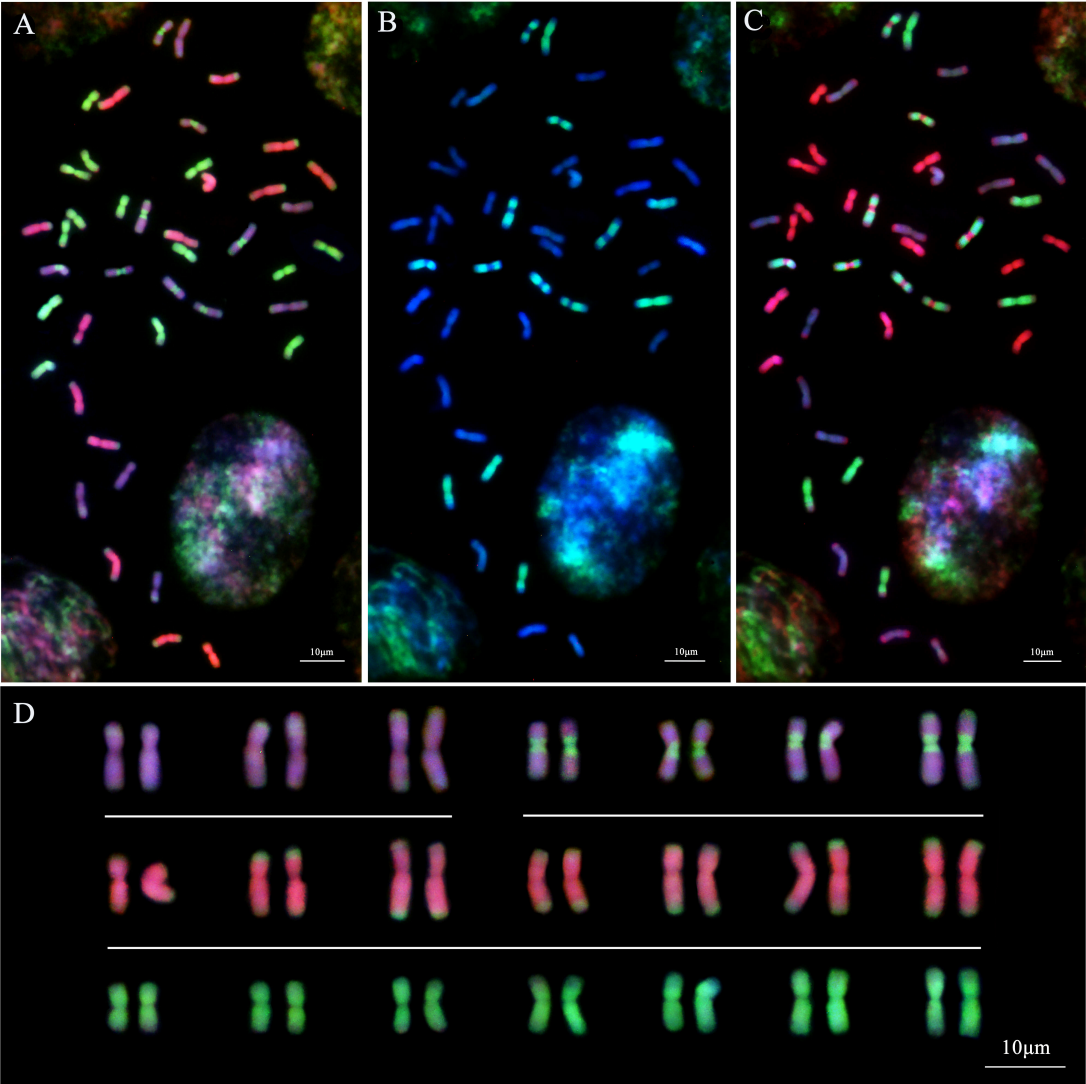
Sequential multicolor GISH analysis of *Th. intermedium* (StStJ^r^J^r^J^vs^J^vs^, 2n=6x=42). **(A)** The *Ps. strigosa* (StSt) probe stains bright-green signals on the entire 14 St-subgenome chromosomes. The *Th. bessarabicum* (J^b^J^b^) probe detects 14 J^r^-subgenome chromosomes with dark-pink signals across the whole chromosomes except for the terminal regions that are covered by the bright-green signals of the St probe. Both St and J^b^ probes display 14 J^vs^ subgenome chromosomes with light-pink signals and bright-green signals. **(B)** The *Dasypyrum villosum* (VV) probe detects 14 J^vs^ subgenome chromosomes with bright-green signals along the entire chromosomes or the whole chromosome arms except the centromeric region. **(C)** The *Ps. strigosa* (StSt) probe (red) labels the same 14 chromosomes, while the *D. villosum* (VV) probe (green) detects 14 J^vs^ subgenome chromosomes together with the St probe. **(D)** The karyotype of *Th. intermedium* based on GISH signals from **(A)**. J^vs^ = Type-I (top left), J^vs^ = Type-II (top right). The chromosomes were counterstained with DAPI (blue). Scale bars = 10 μm.

In the analysis of *Th. intermedium* using the whole genomic DNA from the diploid species *Th. bessarabicum* (J^b^ genome, red) and *Ps. strigosa* (St genome, green) as the probes, 14 St-subgenome chromosomes with complete light-green fluorescent signals were observed in *Th. intermedium* in the first round of GISH analysis (**Figure 6A**). Another 28 chromosomes had the light-green fluorescent signals at the ends or centromeric regions of chromosomes. These light-red fluorescence signals were divided into two types. Fourteen chromosomes produced dark-pink hybridization signals that were spread throughout the long and short arms of chromosomes and had green St-genome probe signals at the end of chromosomes. Another 14 chromosomes developed light-pink hybridization signals. Similarly, there were green St-genome probe signals at the telomeric regions of chromosomes, but six of them were covered by light-pink signals. Besides the telomeric regions, eight chromosomes also had St-genome probe signals at the centromeric regions, and the other parts were covered by a light-pink signal.

When using the whole genomic DNA from the diploid species *D. villosum* (V genome, green) and *Ps. strigosa* (St genome, red) as the probes in the second round of GISH analysis, 14 chromosomes with dark-red signals probed by the J^b^ genomic DNA in the first round of GISH analysis displayed no hybridization signals if the St-genome probe signal channel was turned off. However, 14 chromosomes with the light-pink probed by the J^b^ genome DNA in the first round of GISH displayed the green hybridization signals by the V-genome probe. The positions of the signals on chromosomes were identical to those generated by the J^b^ genome probe. Six chromosomes were completely covered by the V-genome hybridization signals, and eight chromosomes were covered by the V-genome hybridization signals except for the telomeric and centromeric regions (**Figure 6B**). When turning on the signal channel of the St-genome probe, the locations of the hybridization signals were consistent with those of the St-genome probe during the first round of GISH analysis (**Figure 6C**).

The results of the smGISH analyses for IWG showed that 14 chromosomes with complete St-genome probe signals were the St subgenomes of IWG. Fourteen chromosomes were only stained dark-red by the J^b^ genome probe, belonging to the J^r^ subgenome. The remaining 14 chromosomes that showed both light-pink fluorescent signals of the J^b ^genome probe and the V-genome probe signals belonging to the J^vs^ subgenome (**Figure 6C**). According to the hybridization signal patterns, 14 chromosomes of the J^vs^ subgenome were divided into two types. One type, including six chromosomes that were fully covered by the V-genome probed signals except the telomeric regions, were known as Type-I J^vs^ (I J^vs^). The other eight chromosomes, named Type-II J^vs^ (II J^vs^), displayed the St-genome probe hybridization signals at the centromeric and telomeric regions, and the remaining regions of chromosomes were covered by the V-genome probe signals (**Figure 6D**).

The J^b^ genome probe was able to distinguish the J^r^ and J^vs^ subgenomes based on the hybridization signal patterns. However, the V-genome probe only stained the J^vs^ subgenome chromosomes but did not produce any hybridization signals on the J^r^-subgenome chromosomes. The St-genome probe stained not only the St-subgenome chromosomes on the whole arms, but also the J^r^- and J^vs^ subgenome chromosomes at the telomeric and/or centromeric regions. Therefore, the genome of *Th. intermedium* can be expressed as 2n=6x=42 = 14St+14J^r^+14J^vs^ (6 IJ^vs^+8 IIJ^vs^), when probed with the combination of probes from the St- and V-genomic DNA.

### Analysis of chromosome compositions of the best-performed HSC-lines

The smGISH analysis for twenty one 19HSC-Q lines with the same combination of probes found that the chromosome compositions of most lines, for example, line 19HSC-Q2 and 19HSC-21, were the same as IWG, i.e., 2n=6x=42, StStJ^r^J^r^J^vs^J^vs^. However, four of six lines with prominently agronomic traits, 19HSC-Q11, 19HSC-Q14, 19HSC-Q18, 19HSC-Q20, and three 20HSC-Z lines had the translocation and introgression between subgenome chromosomes.

**Figure 7:**
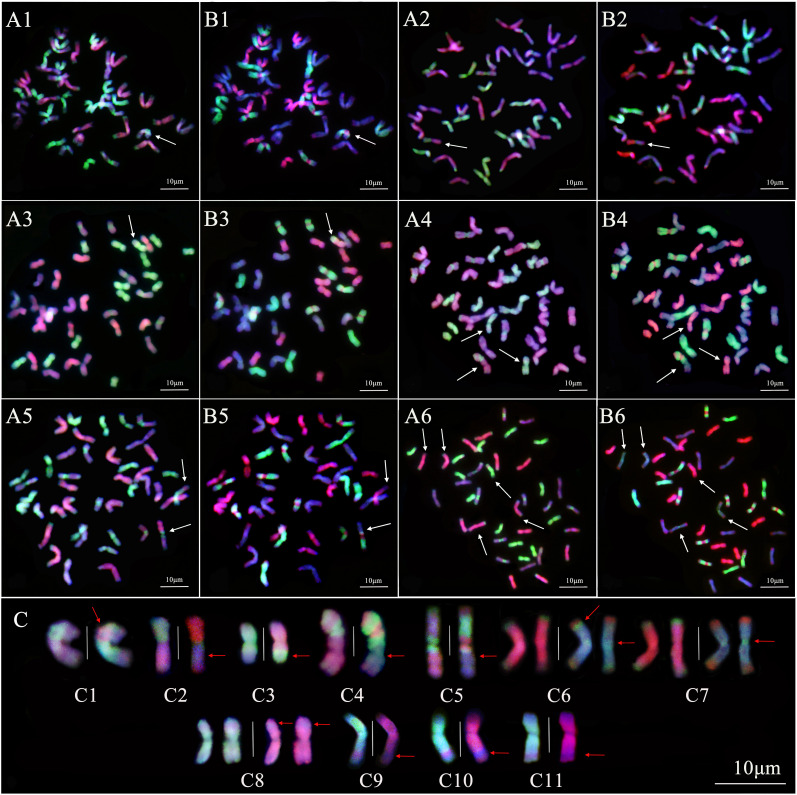
Sequential multicolor GISH analysis of 19HSC-Q lines and 20HSC-Z lines. **(A1–A6)** The probe combination of *Th. bessarabicum* (J^b^ genome, red) and *Ps. strigosa* (St genome, green). **(B1–B6)** The probe combination of *D. villosum* (V genome, green) and *Ps. strigosa* (St genome, red). **(A1, B1)** 19HSC-Q11. **(A2, B2)** 19HSC-Q14. **(A3, B3)** 19HSC-Q18. **(A4, B4)** 19HSC-Q20. **(A5, B5)** 20HSC-Z1. **(A6, B6)** 20HSC-Z2. Signal patterns in both **(A)** and **(B)** are from the same root tip cells. **(C)** Sequential multicolor GISH analysis of the recombinant chromosomes in the 19HSC-Q and 20HSC-Z lines. The white arrows in panels **(A, B)** indicate the recombinant chromosomes. The red arrows indicate the location of chromosome recombinations and the white vertical line is used to separate chromosomes in the panel **(C)**. **(C1)** The introgression of St-IJ^vs^ chromosome; **(C2)** the whole arm translocation of the St-J^r^ chromosome; **(C3)** the terminal translocation of the J^vs^-St chromosome; **(C4)** the terminal translocation of the J^r^-IIJ^vs^ chromosome; **(C5)** the half arm translocation of the J^r^-IIJ^vs^ chromosome; **(C6)** and **(C7)** the introgression of the IJ^vs^-J^r^ chromosome at the terminal parts and the centromeric regions; **(C8)** the small segment translocation of the J^r^-St chromosome at the short arm; and **(C9–C11)** the small segment translocation of the J^r^-St chromosome at the long arm. The chromosomes were counterstained with DAPI (blue). Scale bars = 10 μm.

The pattern of smGISH analysis for line 19HSC-Q11 showed that an IJ^vs^ chromosome had the introgression of St-subgenome chromosomes across the centromeric regions (**Figures 7A1–C1**). Line 19HSC-Q14 showed a chromosome with a bright-green signal of the St genome in the short arm and stained dark-red by the J^b^-genome probe in the long arm. There was no significant green signal of the V-genome probe on the whole arm of the chromosome. It was a whole arm translocation between the short arm of a St chromosome and the long arm of a J^r^ chromosome (**Figures 7A2–C2**). The smGISH analysis of 19HSC-Q18 detected that a St-subgenome chromosome had a large light-green hybridization signal produced by the V-genome probe at the proximal end of the long arm. It was the terminal translocation of a large segment with the J^vs^-St subgenome chromosome (**Figures 7A3–C3**). The smGISH analysis of 19HSC-Q20 identified that a half of the J^r^ chromosome arm was translocated into the end of an IIJ^vs^ chromosome (**Figures 7A4–C4**). In line 20HSC-Z1, a translocation of a half chromosome arm between the J^r^ chromosome and the IIJ^vs^ chromosome was observed (**Figures 7A5–C5**). The introgressions of J^vs^-J^r^ chromosome concentrated on the terminal parts of both long and short arms, as well as the centromeric regions, were found in line 20HSC-Z (**Figures 7C6, C7**). In addition, a small segment of J^r^ chromosome in line 19HSC-Q20 was inserted into a St chromosome at the proximal end of the short arm (**Figures 7A4, B4, C8**). This proximal end small translocation of J^r^-St chromosome was also observed at the long arm in lines 20HSC-Z1 (**Figures 7A5, B5, C9**), 20HSC-Z2 (**Figures 7A6, B6, C10**), and 20HSC-Z3 (**Figure 7C11**).

The results of the smGISH analysis for lines 19HSC-Q and 20HSC-Z detected seven types of chromosome variations based on the size and locus of the translocated fragments. The translocation of large fragments included four types, St-J^r^, J^vs^-St, J^r^-IIJ^vs^, and J^vs^-J^r^, and the small fragment translocations and introgressions contained three types, J^r^-St, St-IJ^vs,^ and J^vs^-J^r^.

## Discussion

The improvement of grasslands is a main approach for the ecological restoration of lightly degraded grasslands, while grassland enclosure and artificial grassland planting are the main methods for the restoration of moderately and extremely degraded grasslands. However, no matter what kind of improvement means. Thereforce, there are high requirements for the forage, including seed yield, cold tolerance, perennial viability, and breeding methods. This is also the direction of modern animal husbandry development. As an important candidate species for perennial crops and forage, *Th. intermedium* has some advantages, such as regeneration, cold-hardiness, and promising grain quality. Nevertheless, the performance of yield components is still far lower than those of annual crops (DeHaan et al., 2020). It is not easy to increase the 1,000-kernal weight and grain numbers per spike through direct domestication of perennial grasses. Wheat*-Th. intermedium* partial amphiploids have better yield component characteristics than *Th. intermedium* per se. After crossing with *Th. intermedium*, it is helpful to introduce wheat genetic components into the genetic background of IWG, so as to improve the yield components of *Th. intermedium*. Therefore, the hybridization between wheat*-Th. intermedium* partial amphiploids and *Th. intermedium* can be used as an efficient way to improve IWG.

Many IWG germplasms have been developed through crossing octoploid trititrigia and *Th. intermedium* since 2006 (Yan et al., 2020). Due to the existence of six subgenomes in the hybrid between tritrigia and IWG, the segregating population of hybrid progenies is massive and it is difficult to identify the candidate plants in the limited breeding time by artificial selection alone. In cold regions, however, perennial crops or forage must be tolerant to cold stress, so that they can be viable in cold winters before resuming growth in spring to complete their growth cycle. We have carried out five selection cycles under the cold winter climate conditions in Harbin. From HNU-1C to HNU-5C, the selected progenies were able to regrow after the cold winter. In this process, the time of florescence and maturity of the HNU-5C plants were about 40 days earlier than that those of HNU-1C and -2C. However, the wheatgrass traits, such as the 1,000-kernal weigh, grain numbers per spike, and tiller number, are still excellent. At similar latitudes, the new *Th. intermedium* variety MN Clearwater bred by the University of Minnesota was also tolerant to cold stress (Cattani et al., 2016). This suggests that it is effective to select perennial crops or wheatgrass in cold winter conditions.

As a perennial forage, IWG cannot be used for pasture improvement on a large scale because of its low yield and seed setting rate (Bajgain and Anderson, 2021). In this study, the grain numbers per spike, seed setting rate, and 1,000-kernal weight of six advanced wheatgrass lines were much higher than those of *Th. intermedium* after five cycles of selection. In particular, grain numbers per spike of these lines exceeded 80, or even >100 in two lines. The 1,000-kernel weights of these lines were above 6 g, and their protein contents were above 17%. These properties were comparable to MN-Clearwater and the derived lines reported by Bajgain et al. (2020). The average seed size of these perennial lines was similar to the MN-series lines. The improvement of these yield component traits will promote the application of perennial wheatgrass in grassland improvement and grassland restoration in the cold regions of the world.

Tiller number and area occupied by individual plant are also the main indicators to evaluate the vitality and yield constitutions of wheatgrass (Altendorf et al., 2021). The tiller number and grain number per spike for line 19HSC-Q were increased over the years. The area occupied by the individual plant was obviously different, spanning a range of 0.080-0.615 m^2^. This demonstrates that tillering and the covered area of perennial grain crops embody the vitality of the underground rhizome in the cold regions. These traits, combined with the performance of yield components, can comprehensively reflect the growth characteristics and genetic differences of plants, as well as the adaptability of plants to the environment. The lines with strong tillering ability and large covered area can be used as forages for the ecological modification and improvement of grasslands in cold regions, as well as low yield fields or poor lands.

The characteristics of a plant phenotype are the result of the combination of genotype and environment. The results of cytogenetic analysis of the *Th. intermedium* genome are controversial, and its classification is also diverse. With the development of genomics technologies, the chromosome composition of *Th. intermedium* has been identified as E^e^E^e^E^b^E^b^StSt or StStJJJ^s^J^s^, in which two subgenomes are closely related with E^e^E^e^E^b^E^b^ or JJJ^s^J^s^, while the other subgenome StSt was far apart (Ji et al., 2001; Chen, 2005). In this study, we used smGISH to analyze IWG with the genomic DNA from St (*Ps. Strigose*), J^b^ (*Th. bessarabicum*), and V (*D. villosum*) as probes and obtained the same results of StStJ^r^J^r^J^vs^J^vs^ (2n=6x=42) as reported by Cseh et al. (2019). According to the fluorescent signals of the J^vs^ subgenome, we divided J^vs^ chromosomes into type-I and type-II, containing three and four pairs of chromosomes, respectively. We found that the J^b^ genome probe could stain and distinguish chromosomes of the J^r^ and J^vs^ subgenomes based on the intensity of hybridization signals. The V-genome probe could only stain the J^vs^ subgenome chromosomes. The signal patterns were consistent with the light color area from the J^b^ genome probe hybridization signals, and no hybridization signals were observed on the J^r^ subgenome chromosomes. This indicates that the J^r^ and J^vs^ subgenomes were closely related, but with obvious differences. Therefore, whether the combination of the St- and J^b^ genome probes or the St- and V-genome probes are used alone, the subgenomes of IWG can be clearly distinguished.

The smGISH analysis of lines in HNU-5C showed that most of the lines had the same chromosomal compositions as IWG, but there were some translocations between the subgenomes of the 19HSC-Q lines and the 20HSC-Z lines, including the insertion of small segment on the short arm of the J^r^-St chromosomes, and the half arm translocation with the J^r^-J^vs^ chromosomes. To better adapt to environments, chromosome recombination occurred during the process of genome rebalancing (Yan et al., 2020). Another explanation is that small translocated or introgressive fragments from wheat chromosomes were unable to be detected through GISH signals due to the high homology between wheat and *Th. intermedium*. This was confirmed by the fact that no obvious genetic components of wheat were detected by the probe of DNA from common wheat cv. Chinese Spring (data not shown). Based on the current results, the observed translocations of subgenome chromosomes existed in some lines with prominently agronomic traits, such as 19HSC-Q11, 19HSC-Q14, 19HSC-Q18, 19HSC-Q20, and 20HSC-Z, which warrants further study to reveal the relationship between chromosomal variations and phenotypes. Meanwhile, the smGISH results provide a new reference for the genomic analysis of wheat-*Th. intermedium* progenies, such as chromosome variations in the synthetic genomes of wheat-*Th. intermedium* partial amphidiploids, as well as the genome selection breeding of IWG.

## Conclusions

Using the domestication method of distant hybridization, we selected six elite lines from 24 new germplasms of *Th. intermedium* at the end of the fifth cycle of selection. The perenniality, cold tolerance, and yield components of these lines were stable for 3 consecutive years and superior to the parental *Th. intermedium* accession. This suggested that the hybrid domestication breeding had improved yield characteristics of *Th. intermedium*. The subgenome chromosome variations of new germplasm enrich the karyotypes of *Th. intermedium*. Some lines with prominently agronomic traits carrying the translocation chromosomes warrant further study to reveal the relationship between chromosomal variations and phenotypes. The smGISH analysis with St, J^b^, and V genome probes provides a new method for further analyzing the relationship between yield traits and genomes of *Th. intermedium*.

## Data availability statement

All data generated or analyzed during this study are included in this published article.

## Author contributions

YL and WWS conceived the project. JD, XNY, JS, ML, XYY, CJ, HZ, and WFS conducted the cold-hardy perennial feature and morphological observation. AS, CW, JD, DL, and XY performed smGISH experiments and the yield components analysis. QS, XL, and LC conducted the statistics analysis. HL and YZ wrote the manuscript. All authors reviewed and approved the manuscript.
